# 3-NOP vs. Halogenated Compound: Methane Production, Ruminal Fermentation and Microbial Community Response in Forage Fed Cattle

**DOI:** 10.3389/fmicb.2018.01582

**Published:** 2018-08-07

**Authors:** Gonzalo Martinez-Fernandez, Stephane Duval, Maik Kindermann, Horst J. Schirra, Stuart E. Denman, Christopher S. McSweeney

**Affiliations:** ^1^CSIRO, Agriculture and Food, Queensland Bioscience Precinct, St. Lucia, QLD, Australia; ^2^Research Centre for Animal Nutrition and Health, DSM Nutritional Products, Saint-Louis, France; ^3^Animal Nutrition and Health, DSM Nutritional Products, Basel, Switzerland; ^4^The University of Queensland, Centre for Advanced Imaging, Brisbane, QLD, Australia

**Keywords:** rumen, 3-NOP, chloroform, methane, microbiota, methyl compounds, NMR

## Abstract

The aim of this study was to investigate the effects of 3-nitrooxypropanol (3-NOP) and chloroform on methane (CH_4_) and H_2_ production, ruminal metabolites and microbial community structure in cattle fed a tropical forage diet. Eight rumen-fistulated steers were fed a roughage hay diet (Rhodes grass; *Chloris gayana*) for 31 days (control period). Four animals received the antimethanogenic compound chloroform (1.6 g chloroform–cyclodextrin/100 kg live weight (LW)) while the other four received 3-NOP (2.5 g 3-NOP/animal/day) for 21 days. Methane decrease compared with control period was similar for both treatments (30–38%) with no differences for expelled H_2_ between controls and treatments. Daily weight gain (DWG) was significantly increased when animals were treated with 3-NOP compared with chloroform and control. Regarding the ruminal fermentation parameters increases in ammonia, acetate and branched chain fatty acids were observed with both compounds compared with the controls. Also, methylamines, alcohols and dimethyl sulfone (DMSO_2_) concentrations were significantly increased with the treatments compared with control, being greater with 3-NOP. The rumen microbial analyses revealed a similar profile for both treatments, with a shift in operational taxonomic units (OTUs) assigned to the Prevotellaceae and Campylobacteraceae family. Moreover, major archaeal OTUs associated with *Methanobrevibacter* and *Methanosphaera* were significantly affected to varying extents based on the inhibitory treatments compared to the control. The abundance of the *Methanobrevibacter* spp. was decreased by 3-NOP and chloroform, while the Methanomassiliicoccaceae family was inhibited only by 3-NOP. The results suggest that despite the specific mode of action of 3-NOP on methanogens, inhibition of methanogenesis by both compounds resulted in similar responses in metabolism and microbial community structure in the rumen. We hypothesized that these changes were driven by the redirection of metabolic hydrogen ([H]) by both treatments. Therefore results from previous publications using chloroform as an inhibitor of methanogenesis may be useful in predicting ruminal microbiota and fermentation responses to 3-NOP.

## Introduction

Over the last decade, methane (CH_4_) production by livestock has been targeted by animal nutritionists because of its significant contribution to anthropogenic greenhouse gases emissions (Gerber et al., 2013). Methane has a greenhouse gas warming potential 28× greater than CO_2_, and ruminants are responsible for ∼20% of global CH_4_ emissions (Conrad, 2005). In the rumen, CH_4_ is the major gaseous by-product generated by methanogens during the fermentation of feed, and represents a significant loss of gross feed energy (2–12%) for ruminants (Johnson and Johnson, 1995).

A wide range of strategies, such as the use of CH_4_ inhibitors, vaccines and dietary management, have shown variable results when tested in ruminants to decrease CH_4_ formation. Among the synthetic CH_4_ inhibitors, halogenated compounds have been used due to their effectiveness although they can be toxic or cause a negative impact on the environment themselves, and are therefore unsuitable for use as practical feed additives (Gerber et al., 2013). However, the use of chloroform as a model for directly inhibiting methanogens and examining the response in metabolic hydrogen [H] flow and rumen microbial community structure in cattle has provided new insights into ruminal metabolism (Martinez-Fernandez et al., 2016, 2017). These studies showed that when chloroform decreased CH_4_ formation by about 30% there were no apparent detrimental effects on the broader rumen microbial ecology in cattle fed a tropical grass hay alone or supplemented with concentrate. Also, it was observed that there was relatively less expelled H_2_ for the hay diet suggesting a more efficient redirection of [H] into other microbial end-products compared with the concentrate diet. The mechanism of action of chloroform has not been confirmed, although it appears to interfere at the cobamide-dependent methyl transferase step of the methanogenesis pathway but there could also be collateral inhibition of methyl transferases in other bacteria (Gunsalus and Wolfe, 1978; Graham and White, 2002).

Recently, a synthetic non-toxic compound, 3-nitrooxypropanol (3-NOP), has been developed which consistently decreases CH_4_ production in a range of small and large ruminant trials and thus shows promise as a commercial feed additive (Haisan et al., 2014, 2016; Martinez-Fernandez et al., 2014; Romero-Perez et al., 2014; Hristov et al., 2015; Vyas et al., 2016a,b, 2018). Also, some studies have reported an increase in daily weight gain (DWG) in dairy cows (Haisan et al., 2014; Hristov et al., 2015). The compound specifically inhibits methanogens by binding to the active site of the methyl-coenzyme M reductase enzyme that performs the terminal reaction of the methanogenesis pathway (Duin et al., 2016). However, all the feeding studies with 3-NOP have focused on high quality concentrate-based diets, and there is no information on the effectiveness of the compound in cattle fed lower quality roughage.

Thus, the aim of this trial was to compare the effect of 3-NOP with chloroform on CH_4_ and H_2_ production, ruminal metabolites and microbial community structure in cattle fed a tropical hay diet. It was hypothesized that a moderate decrease in CH_4_ (∼30%) due to either compound would not have an adverse effect on ruminal metabolism and a similar shift in rumen microbial populations would occur as a consequence of the redirection of [H] away from CH_4_ formation to other metabolic sinks in the rumen.

## Materials and Methods

The experimental protocol complied with the Australian Code for the Care and Use of Animals for Scientific Purposes (eighth edition, 2013) and was approved by the local Animal Experimentation and Ethics Committee (A08/2014).

### Experimental Design and Sampling

Eight rumen-fistulated Brahman steers (*Bos indicus*, live weight (LW) 490 ± 10 kg and 3 years old) at Lansdown Research Station (Townsville, QLD, Australia) were used in the current study. Animals were randomly allocated to two groups (four animals per group (**Supplementary Figure S1**), LW 492 ± 7 kg and 488 ± 13 kg for each group) and weighed every 21 days prior feeding during the trial duration. The experimental diet used was a tropical forage offered *ad libitum* (Rhodes grass hay, *Chloris gayana*), chemical composition: DM 917 g/kg fresh matter; OM 806; CP 169; NDF 661; ADF 359; ADL 46; ash 116 and GE 17.38 MJ/kg. The treatments used were chloroform encapsulated in cyclodextrin (6–7% w/w chloroform, as described by Martinez-Fernandez et al. (2016)) and 3-NOP (DSM Nutritional Products, Ltd., Basel, Switzerland) mixed with 60 mL/day of molasses (molasses was diluted in water, 1:4 water:molasses).

Animals were adapted to the diet over a 31 days period, with the last 10 days of the period placed into individual pens for the measurement of intakes and treated with cyclodextrin (1.6 g/100 kg LW) and molasses (60 mL/day). On the last 2 days of that period animals were placed into open-circuit respiration chambers for measurement of CH_4_ and H_2_ production and collection of rumen samples. Following the initial control period one group of animals received through the cannula the chloroform for 21 days (1.6 g chloroform–cyclodextrin/100 kg LW divided in two shots at 0 h and 6 h after feeding) (Martinez-Fernandez et al., 2016) and the second group received the 3-NOP treatment for 21 days (2.5 g 3-NOP/animal/day). The 3-NOP (mixed with molasses) was provided to the animals mixed with the hay at three different times: 0 h, 3 h and 6 h after the feed was offered in an attempt to extend the exposure of the compound to the rumen which occurs with cyclodextrin–chloroform complex. The 3-NOP and chloroform groups were treated with comparative amounts of cyclodextrin or molasses, respectively. On days 20 and 21 of treatment both groups were placed in open-circuit respiration chambers for direct measurement of CH_4_ and H_2_ production and rumen fluid collection.

Rumen fluid samples (approx. 60 mL/animal) were collected using a probe with two layers of cheesecloth through the cannula of the animal at 3 h post feeding, during confinement in respiration chambers to determine the effect on ruminal fermentation parameters and rumen microbial communities. Rumen samples were placed on dry ice and stored at −20°C for ruminal fermentation metabolites analyses. Additionally, several rumen samples were centrifuged (13,000 ×*g* for 5 min), and the supernatant was removed before placing on dry ice and later storing at −80°C prior to DNA extractions for microbial community composition. In addition, when animals were placed in chambers, samples of Rhodes grass hay placed in nylon bags were incubated in the rumen of steers to determine DM ruminal degradation over 24 h and 48 h.

### Gas Measurements

Four open-circuit respiration chambers were used to determine CH_4_ and H_2_ production from individual steers as described by Martinez-Fernandez et al. (2016). Briefly, CH_4_ and H_2_ emissions were performed using a combination of negative pressure (–10.1 ± 0.14 Pa) in four clear polycarbonate and independent units (23.04 m^3^, 3000 L/min air flow). Air samples passed through a chemical drier and were metered through independent rotameters before compositional analysis for CH_4_ (Servomex 4100 Servomex Group Ltd., Crowborough, United Kingdom) and H_2_ (Servomex Chroma, Servomex Group Ltd., Crowborough, United Kingdom; and Dräger X-am 5000, Draeger Safety Pacific Pty. Ltd., Notting Hill, VIC, Australia). CH_4_ and H_2_ production (g) were calculated by averaging 48 h measurements.

### Chemical Analysis

Feed samples were dried in a forced-air oven at 105°C prior to grinding. Feed samples were ground through a 1 mm sieve before analysis. DM, ash, NDF, ADF, lignin, ether extract, gross energy (adiabatic calorimeter) and total nitrogen contents were analyzed by Symbio Alliance (Eight Mile Plains, QLD, Australia) following the accredited methods CF006.1 (925.10), CF007 (923.03), CF038.2 (973.18), CF038.3 (2002.04), CF038.6 (973.18), CF004.1 (920.39), CF237 and CF003.2 (990.03), respectively (Association of Official Analytical Chemists [AOAC], 2005. Official methods in parenthesis). The nitrogen values were converted to CP by a factor of 6.25. A constant energy value of 55.22 MJ/kg CH_4_ (Brouwer, 1965) was adopted for calculation of the CH_4_ conversion rate (Ym) which estimates the fraction of GE an animal consumes that is converted to CH_4_.

Concentrations of short chain fatty acids (SCFAs) (acetate, propionate, *n*-butyrate, iso-butyrate, iso-valerate and *n*-valerate) were measured by gas chromatography (GC) as described by Gagen et al. (2014). Iso-valerate (3-methyl butyrate) includes 2-methylbutyrate, which co-elutes. The SCFAs were quantified by NMR as well and yielded similar results (data not shown).

The NH_3_–N concentration was determined by a colorimetric method previously published (Chaney and Marbach, 1962).

### Ruminal Degradability of Rhodes Grass Hay

Ruminal degradability was measured for 3 g of 2 mm ground hay. Samples were placed in 5 cm × 10 cm nylon bags with a pore size of 50 μm. Four bags were incubated in the rumen of each steer immediately before the morning feeding. Two bags were withdrawn after 24 h and the other two after 48 h of incubation. Blank bags were used for accounting for losses of bag materials. At the end of the corresponding incubation times bags were washed with cold water and maintained at −20°C before being washed in a washing machine using a short cold water program including two bags with feed that had not been incubated in the rumen to account for solubility. After washing, bags were placed in the oven at 60°C for 48 h. Ruminal degradability was calculated as the loss of dry matter over the corresponding incubation time.

### Rumen Metabolomics Analyses

Samples from rumen fluid were thawed on ice, and 240 μL of ruminal fluid was mixed with 60 μL of potassium phosphate buffer (pH 7.4), containing NaN_3_ as a preservative, D_2_O as a field lock, 4,4-dimethyl-4-silapentane-1-sulfonic acid (DSS) as an internal chemical shift standard, and 1,1-difluoro-1-trimethylsilanyl methylphosphonic acid (DFTMP) as an internal pH indicator, leading to final concentrations of 143 mM phosphate buffer, 0.05% (w/v) NaN_3_, 9.5% (v/v) D_2_O, and 95.2 M DSS and DFTMP. Precipitates were removed by centrifugation (13,000 × *g*, 15 min, 4°C), and 200 μL of the supernatant was transferred into 3 mm NMR tubes.

^1^H NMR spectra were recorded on a Bruker AV900 NMR spectrometer (Bruker Biospin, Rheinstetten, Germany) operating at a ^1^H frequency of 900.13 MHz and equipped with a 5 mm self-shielded z-gradient triple resonance probe and a SampleJet sample changer chilled to 6°C. For each rumen sample a 1D NOESY spectrum was acquired at 298 K with the *noesypr1d* pulse sequence ((RD)–90°–t1–90°–τ_m_–90°–acq.) (Bruker Biospin pulse program library, Germany). The transmitter frequency was set to the frequency of the water signal, and water suppression was achieved by continuous wave irradiation during both the relaxation delay of 4.0 s and the mixing time (τ_m_) of 100 ms. After eight dummy scans, 128 transients were collected into 131,072 data points using a spectral width of 20.0 ppm. All spectra were processed using TOPSPIN version 3.2 (Bruker Biospin, Rheinstetten, Germany). The free induction delays (FIDs) were multiplied by an exponential function corresponding to a line broadening of 0.3 Hz before Fourier transformation, manually phase and baseline correction. The resulting spectra were referenced to the DSS signal at *δ* = 0 ppm. The assignment of peaks to specific metabolites (**Supplementary Table S1**) was based on identification in Chenomx NMR Suite 8.2 (Chenomx Inc., Edmonton, AB, Canada) as well as the online Rumen Metabolome Database (Saleem et al., 2013).

To correct for slight differences in peak alignment, ^1^H NMR spectra were aligned in MATLAB (The Mathwork, Natick, MA, United States) with the icoshift algorithm (Savorani et al., 2010), and then automatically data-reduced to consecutive integral regions of 0.001 ppm width (“buckets”), covering the range of *δ* = 10.0 – 0.3 ppm. The chemical shift region at *δ* 5.0 – 4.6 ppm was excluded to eliminate effects of imperfect water suppression. For each spectrum, the resulting integral regions were normalized to the total spectrum intensity to correct for inter-sample differences in dilution.

The bucketed 1D NOESY NMR spectra were initially subjected to untargeted multivariate statistical analysis in the SIMCA-P + 12.0 software package (Umetrics AB, Sweden). This type of analysis reveals systematic changes in the intensity of any signal in the NMR spectra. These signals originate from aqueous metabolites of core metabolism that are present in at least micromolar concentrations. Identification data for specific metabolites are listed in **Supplementary Table S1**. A Pareto-scaled principal components analysis (PCA) was performed to investigate inherent sample differences among all sample classes, as well as between treatment and controls and between both treatments (**Supplementary Figure S2**). The number of latent components in each model was optimized by cross validation. *R*^2^*X* and *Q*^2^ were used to evaluate model quality. *R*^2^*X* is the fraction of the sum of squares for the selected component representing the variance of the *X* variables, and *Q*^2^ is the predictive ability parameter of the model, which is estimated by cross validation. The figures of merit of all multivariate models are listed in **Supplementary Table S2**.

Metabolites that were markedly changing between the individual groups (treatment-control or between treatments) were identified from the loadings plot, and the resulting 29 metabolites (listed in **Supplementary Figure S3E**) were quantified in each spectrum with Chenomx NMR Suite 8.2. The table of quantified metabolite data was subsequently analyzed in a further round of targeted PCA (Pareto-scaled) for systematic metabolite differences (**Supplementary Figure S3**). As the name targeted suggests, in this analysis the concentration changes in only these 29 metabolites were characterized in a focused manner, whereas the previous untargeted analysis investigated any intensity changes in the NMR spectra, independent of subsequent metabolite identification.

### 16S rRNA Gene Analysis

DNA extractions from rumen pellet samples were performed as previously described employing a bead beating method for lysis (Martinez-Fernandez et al., 2016). The yield and purity of the extracted DNA were assessed with a NanoDrop 8000 spectrophotometer (Thermo Fisher Scientific, Wilmington, DE, United States). The 16S rRNA gene was used to characterize the microbial populations present in the rumen for the control and treatment periods. The V4 region of the 16S rRNA gene was targeted using specific primers (Kozich et al., 2013). Each individual DNA sample was amplified using the specific primers and a unique barcode combination. Amplification products were visualized by performing gel electrophoresis. Product quantities were calculated and an equal molar amount of each product was pooled. The pooled products were run in a 1% agarose gel and bands were visualized and excised under blue light trans-illumination. The amplicons were gel purified with QIAquick Gel extraction Kit (Qiagen, Hilden, Germany) prior to submission for Illumina Miseq sequencing (Macrogen Inc., South Korea).

Paired end short read sequence data generated on the Illumina Miseq was processed using the USEARCH package (Edgar, 2010). De-multiplexed paired end sequences were first merged prior to sequence quality filtering, followed by denoising (error correction) and chimera checking and clustering of sequences to operational taxonomic units (OTUs) of 97% similarity. Taxonomic assignment of sequences was performed against the Greengenes database (McDonald et al., 2012). Additional analysis of OTUs was performed in the R packages vegan, Phyloseq, DESeq2 and the ggplot2 graphics package (McMurdie and Holmes, 2013; Oksanen et al., 2013; Love et al., 2014; Wickham, 2016). The significances of grouping in the PCoA plots were tested by analysis of dissimilarity (ADONIS) with 999 permutations. The sequences obtained in this paper have been deposited in the European Nucleotide Archive under the accession number PRJEB24539.

### Real-Time PCR Analysis

The DNA samples were used as templates for quantifying the abundance of the *mcrA* gene for total methanogens, and the 16S rRNA for *Methanobrevibacter* and Methanomassiliicoccaceae family specific. The primers and assay conditions used were previously published by Denman et al. (2007) and Huang et al. (2016). Real-time PCR (qPCR) analyses were run in quadruplicate from one DNA extraction on an Applied Biosystems^TM^ ViiA^TM^ 7 Real-Time PCR System (Thermo Fisher Scientific Inc.). Assays were set up using the SensiFAST SYBR^®^ Lo-ROX reagents (Bioline). Optimization of assay conditions was performed for primer, template DNA and MgCl_2_ concentrations. An optimal primer concentration of 400 nM, with a final MgC_l2_ concentration of 3 mM and DNA template concentration of 50 ng were used for each assay under the following cycle conditions: one cycle of 50°C for 10 s and 95°C for 2 min 30 s for initial denaturation, 40 cycles at 95°C for 15 s and 60°C for 1 min for primer annealing and product elongation. Fluorescence detection was performed at the end of each annealing and extension step. Amplicon specificity was performed via dissociation curve analysis of PCR end-products by raising the temperature at a rate of 0.05°C/s from 60 to 95°C. Changes in targeted populations were calculated using a relative quantification calculation and the 2^−ΔΔCt^ method, with the control period used as the calibrator and total bacterial ct (cycle threshold) values used as the reference value (Livak and Schmittgen, 2001; Denman and McSweeney, 2006).

### Statistical Analyses

To study the pre-treatment effect and account for the time effect, data from 3-NOP, chloroform and their respective control periods were analyzed separately as a univariate repeated-measures analysis of variance using the GLM procedure of SPSS (IBM, version 21.0). The effect of treatment was analyzed for CH_4_ and H_2_ production, dry matter intake (DMI), LW, DWG, ruminal fermentation metabolites and methanogen abundances. To study the effect of the compounds after 21 days of treatment, chloroform and 3-NOP groups were compared as a univariate model using the GLM procedure of SPSS, the treatment was considered the fixed effect with the animal as experimental unit and the pre-treatment data as covariate. The effect of the treatment was analyzed for CH_4_ and H_2_, DMI, LW, DWG, ruminal fermentation metabolites and methanogens abundances. Effects were declared significant at *P* ≤ 0.05 and *P*-values between 0.05 and 0.10 were considered as a trend.

## Results

### Ruminal Fermentation and Gas Production

Dry matter intake, CH_4_ and H_2_ production and percent gross energy intake (GEI) lost as CH_4_ were not significantly different between treatments (**Table 1**). The DWG and LW significantly increased when animals were treated with 3-NOP compared with the chloroform group. Regarding the fermentation parameters, no significant effects were observed between 3-NOP and chloroform for the SCFA profile and ammonia concentrations, with only a small significant increase in pH with 3-NOP (**Table 1**).

**Table 1:** Chloroform and 3-NOP effects on CH_4_ and H_2_ production, dry matter intake (DMI), live weight (LW), daily weight gain (DWG) and rumen fermentation parameters in steers fed at forage diet (Rhodes grass hay).

		Chloroform	3-NOP	SEM^c^	*P-value*
DMI in pens (kg)	7.4	7.7	0.12	0.49
DMI in chambers (kg)	6.7	7.4	0.22	0.13
LW (kg)	496	499	6.41	0.008
DWG (kg)	−0.060	0.571	0.07	0.008
CH_4_ (g/d)	109	104	3.58	0.49
CH_4_ (g/kg DMI)	16.3	14.2	0.56	0.20
H_2_ (g/day)	0.003	0.004	0.0003	0.09
Ym *(%)^a^*	5.2	4.5	0.18	0.20
pH	6.73	6.96	0.03	0.027
DM degradability % (24 h)^b^	30.0	31.3	0.74	0.33
DM degradability % (48 h)^b^	39.0	38.1	0.46	0.63
NH_3_-N (mg/100 mL)	28.1	27.8	1.43	0.87
Total SCFA, (mM)	94.7	86.6	3.92	0.12
Individual SCFA (mol/100 mol)				
Acetate	74.1	74.4	0.23	0.11
Propionate	16.3	15.9	0.25	0.45
i-Butyrate	1.3	1.3	0.04	0.55
Butyrate	4.9	5.0	0.27	0.71
i-Valerate	1.7	1.7	0.06	0.60
Valerate	1.4	1.5	0.03	0.26
Caproate	0.3	0.1	0.01	0.83
A:P	4.5	4.7	0.07	0.31
Ruminal metabolites by NMR (μmol/L rumen fluid)				
Lactate	30.7	13.3	1.29	0.001
Benzoate	17.4	12.2	0.79	0.034
Phenyl acetate	203	160	11.2	0.06
Dimethyl sulfone	80	113	4.57	0.011
Trimethylamine	827	1242	105	0.046
Methanol	226	118	9.39	0.001

*^a^Methane conversion rate (Ym) = % GEI lost as CH_4_. ^b^In sacco degradability of Rhodes grass hay. ^c^SEM, standard error of the mean.*

The comparisons between control periods and treatments are shown in **Tables 2**, **3**. Dry matter intakes significantly increased when animals were treated with 3-NOP, compared with the control period. Methane production (g) per kg of DMI was decreased significantly by 3-NOP (38%) and chloroform (30%) compared with their respective control, consequently the loss of energy as CH_4_ decreased significantly with the treatments (from 7.4% in controls to 4.5% and 5.2% for 3-NOP and chloroform, respectively). Surprisingly, no significant differences in the amount of expelled H_2_ were observed between controls and treatments. Daily LW gain only increased significantly for the 3-NOP treatment. A similar pattern in fermentation parameters was observed with both compounds compared with their respective control periods, with a significant increase in relative concentrations of acetate and branched chain fatty acids, and a decrease in butyrate and caproate. Ammonia concentration increased with both treatments compared with their control periods, although just a trend was observed with 3-NOP. Although the acetate: propionate ratio was unchanged by the treatments compared with control periods 3 h after feeding, a significant decrease (from 4.52 to 4.02) for chloroform and a numerical (*P* = 0.146) decrease (from 4.71 to 4.14) by 3-NOP were observed in rumen samples collected prior to feeding (**Supplementary Tables S3, S4**).

**Table 2:** The 3-NOP effects compared with control period on CH_4_ and H_2_ production, dry matter intake, live weight, daily weight gain and fermentation parameters in steers fed at forage diet (Rhodes grass hay).

	Control	3-NOP	SEM^d^	*P-value*
DMI in pens (kg)	7.7	7.6	0.22	0.32
DMI in chambers (kg)	6.6	7.4	0.37	0.035
LW (kg)	487	499	9.21	0.003
DWG (kg)^a^	−0.040	0.571	0.09	0.012
CH_4_ (g/d)	149	104	1.38	0.016
CH_4_ (g/kg DMI)	22.9	14.2	1.0	0.005
H_2_ (g/day)	0.004	0.004	0.001	0.77
Ym *(%)*^b^	7.3	4.5	0.32	0.005
pH	6.87	6.96	0.06	0.71
DM degradability % (24 h)^c^	33.7	31.3	0.49	0.049
DM degradability % (48 h)^c^	40.4	38.1	0.42	0.07
NH_3_-N (mg/100 mL)	22.3	27.8	2.13	0.06
Total SCFA, (mM)	102	86.6	8.7	0.12
Individual SCFA (mol/100 mol)				
Acetate	73.5	74.4	0.38	0.016
Propionate	16.1	15.9	0.33	0.60
i-Butvrate	0.9	1.3	0.07	0.039
Butvrate	6.7	5.0	0.42	0.009
i-Valerate	1.2	1.7	0.05	0.001
Valerate	1.2	1.5	0.07	0.11
Caproate	0.37	0.23	0.02	0.001
A:P	4.6	4.7	0.11	0.28
Ruminal metabolites by NMR				
(μmol/L rumen fluid)				
Glucose	24	17	4.54	0.49
Maltose	11	5.6	1.13	0.15
Phenyl acetate	134	159	12.9	0.16
Dimethyl sulfone	20	113	3.84	0.001
N,N-Dimethylglycine	31	87	19.1	0.21
Trimethylamine	334	1242	137	0.016
Dimethylacetamide	133	321	34.5	0.029
Ethanol	49	54	4.02	0.10
Methanol	70	118	17.5	0.14

*^a^DWG for the control period corresponds to the period of time where the animals were adapted to the diet (30 days), ^b^Methane conversion rate (Ym) = % GEI lost as CH_4_. ^c^In sacco degradability of Rhodes grass hay. ^d^SEM, standard error of the mean.*

**Table 3:** Chloroform effects compared with control period on CH_4_ and H_2_ production, dry matter intake, live weight, daily weight gain and rumen fermentation parameters in animals fed at forage diet (Rhodes grass hay).

	Control	Chloroform	SEM^d^	*P-value*
DMI in pens (kg)	7.6	7.4	0.17	0.41
DMI in chambers (kg)	7.0	6.7	0.39	0.60
LW (kg)	497	496	7.5	0.70
DWG (kg)^a^	0.183	−0.060	0.14	0.19
CH_4_ (g/d)	162	109	5.24	0.012
CH_4_ (g/kg DMI)	23.2	16.3	0.54	0.001
H_2_ (g/day)	0.004	0.003	0.001	0.45
Ym *(%)*^b^	7.4	5.2	0.17	0.001
pH	6.76	6.73	0.05	0.38
DM degradability % (24 h)^c^	32.7	30.0	0.90	0.20
DM degradability % (48 h)^c^	39.3	38.9	0.38	0.47
NH_3_-N (mg/100 mL)	8.8	28.1	1.7	0.007
Total SCFA, (mM)	94.7	94.7	3.2	0.99
Individual SCFA (mol/100 mol)				
Acetate	70.0	74.1	0.55	0.029
Propionate	16.6	16.3	0.42	0.59
i-Butvrate	0.64	1.3	0.06	0.008
Butvrate	9.6	4.9	0.23	0.001
i-Valerate	1.15	1.7	0.10	0.06
Valerate	1.4	1.4	0.09	0.83
Caproate	0.65	0.30	0.04	0.004
A:P	4.2	4.5	0.13	0.17
Ruminal metabolites by NMR				
(μmol/L rumen fluid)				
Glucose	32	15	2.83	0.001
Maltose	13	5	0.63	0.002
Phenyl acetate	69	203	5.63	0.003
Dimethyl sulfone	21	80	3.85	0.007
N,N-Dimethylglycine	37	91	9.05	0.005
Trimethylamine	564	828	100	0.025
Dimethyl acetami de	282	217	35.5	0.43
Ethanol	63	146	19.5	0.14
Methanol	54	226	19.8	0.009

*^a^DWG for the control period corresponds to the period of time where the animals were adapted to the diet (30 days). ^b^Methane conversion rate (Ym) = % GEI lost as CH_4_. ^c^In sacco degradability of Rhodes grass hay. ^d^SEM, standard error of the mean.*

The CH_4_ emissions pattern through the day (**Supplementary Figure S4**) consisted of larger differences among treatments over the first 8 h after feeding, and then they gradually came closer toward the end of the day. When animals were treated with chloroform, CH_4_ emissions were decreased more consistently than 3-NOP treatment, probably due to the administration and delivery methods used.

The ruminal DM degradability of the Rhodes grass hay at 24 h of incubation slightly decreased with 3-NOP but not with the chloroform compared with the control period (**Tables 2**, **3**). However, no significant differences were observed between treatments (**Table 1**).

Ruminal metabolites (μmol/L rumen fluid) that were significantly different for control and treated animals are shown in **Tables 2**, **3**. Some methylamines (trimethylamine and dimethylacetamide) and other metabolites (dimethylsulfone, DMSO_2_) significantly increased with 3-NOP treatment compared with control period. Regarding the chloroform treated-animals a significant increase in methanol, *N*,*N*-dimethylglycine and phenylacetate and a decrease in glucose and maltose were observed compared with the control period. On the other hand, dimethylsufone, lactate, trimethylamine, methanol and benzoate concentrations were significantly different between treatments, with an increase of DMSO_2_ and trimethylamine and a decrease of lactate, methanol and benzoate in 3-NOP compared with chloroform treated animals (**Table 1**).

### Microbial Community

The diversity analysis of the ruminal microbiota showed a significant contraction in Shannon and Simpson diversity for both treatments compared with control (**Supplementary Figure S5**). The structure of the microbiota as determined by non-phylogenetic (Bray-Curtis) and phylogenetic (weighted and unweighted Unifrac) beta diversity analyzes showed a significant separation (ADONIS) between the control and treatments. Beta dispersions were non-significant for the Bray-Curtis and weighted Unifrac (*P* ≤ 0.05) analyzes, whilst it was significant for the unweighted Unifrac. (**Supplementary Figure S6**).

The ratios of sequences assigned to Archaea and Synergistetes in relation to bacteria, decreased (*P* ≤ 0.01) with 3-NOP and chloroform treatments (**Supplementary Figure S7**). The ratio of hydrogenotrophic methanogens (*Methanobrevibacter* genus) to methylotrophic methanogens (Methanomassiliicoccaceae and *Methanosphaera*) decreased (*P* ≤ 0.05) with both compounds compared with the control, which suggested a greater effect of the compounds on the hydrogenotrophic methanogens. However, chloroform showed a greater negative effect on *Methanosphaera* genus, with a significant decrease in the relative abundance of *Methanosphaera* compared with the control and 3-NOP, whilst 3-NOP showed a significant decrease in *Methanomassiliicoccus* genus. On the other hand, no significant differences were observed in *Ruminococcus* and Fibrobacteres relative abundances between the treatments and control.

**FIGURE 1:**
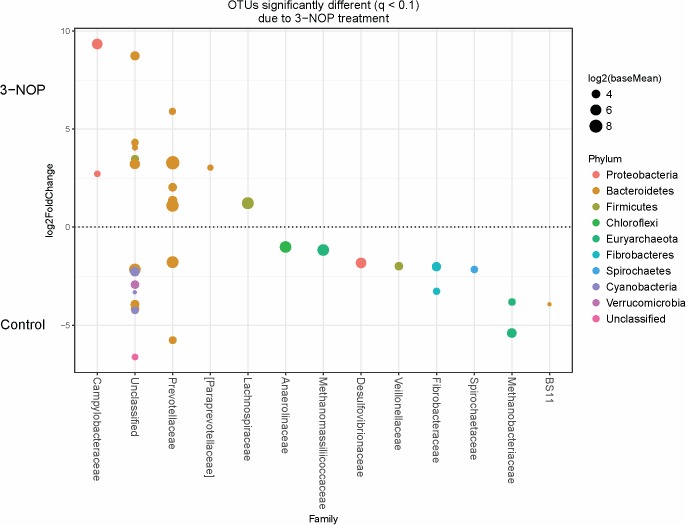
Operational taxonomic units (OTUs) significantly different (*q* ≤ 0.1 FDR) between 3-NOP treated-steers and control period. Upper axis represents OTU’s with a log2 fold positive difference for 3-NOP treatment relative to control while the lower y-axis is the negative fold difference of the 3-NOP relative to control. Each point represents a single OTU colored by phylum and grouped on the x-axis by taxonomic family level, size of point reflects the log2 mean abundance of the sequence data.

**FIGURE 2:**
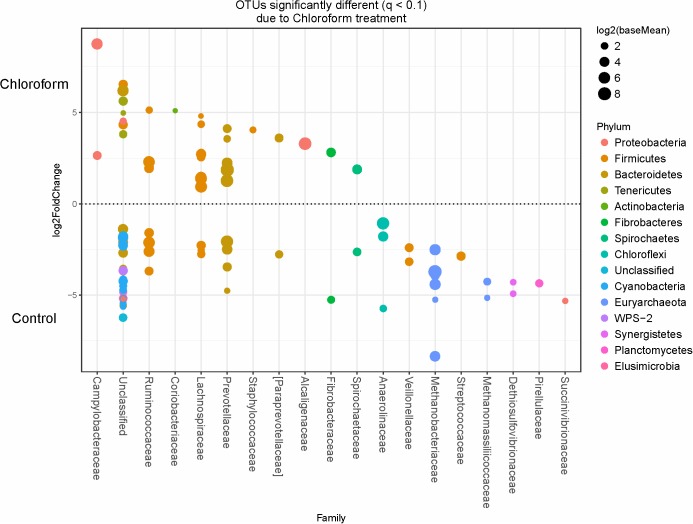
OTUs significantly different (*q* ≤ 0.1 FDR) between chloroform treated-steers and control period. Upper axis represents OTU’s with a log2 fold positive difference for chloroform treatment relative to control while the lower y-axis is the negative fold difference of the chloroform relative to control. Each point represents a single OTU colored by phylum and grouped on the x-axis by taxonomic family level, size of point reflects the log2 mean abundance of the sequence data.

**FIGURE 3:**
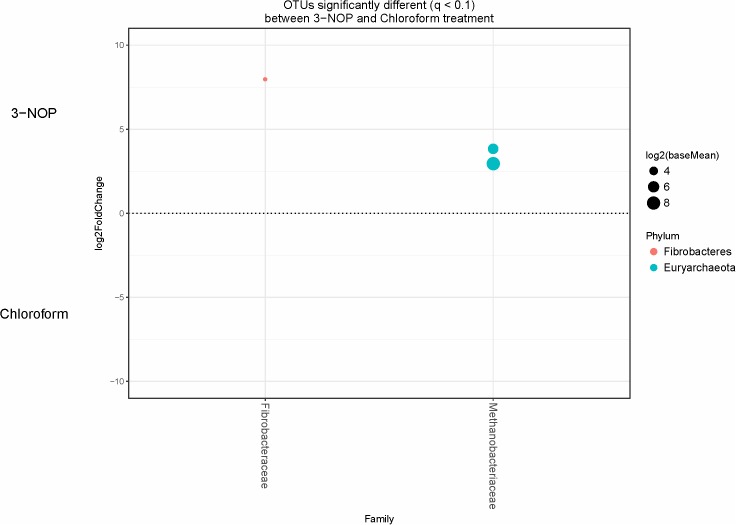
OTUs significantly different (*q* < 0.1 FDR) between 3-NOP and chloroform treated-steers. Upper axis represents OTU’s with a log2 fold positive difference for 3-NOP treatment relative to chloroform while the lower y-axis is the negative fold difference of the 3-NOP relative to chloroform. Each point represents a single OTU colored by phylum and grouped on the x-axis by taxonomic family level, size of point reflects the log2 mean abundance of the sequence data.

Specific bacterial OTUs that were significantly increased with the 3-NOP and chloroform treatments compared with controls (**Figures 1**, **2**) were classified within the Prevotellaceae and Campylobacteraceae family. However, relative to the controls the 3-NOP and chloroform treatments were also negatively associated with the abundance of some other OTUs assigned to *Prevotella* genus. A shift between OTUs within the Ruminococcaceae and Lachnospiraceae families were observed only with the chloroform and its control. Minor OTUs assigned to Fibrobacteraceae, Veillonellaceae, Anaerolinaceae and Spirochaetaceae families were decreased with both compounds. Both compounds decreased the relative abundance of OTUs classified within the Methanobacteriaceae and Methanomasiliicoccaceae families (**Figures 1**, **2**), although OTUs assigned to *Methanosphaera* genus declined more with the chloroform (data not shown) compared to control. When both treatments were directly compared, only some OTUs within the Methanobacteriaceae and Fibrobacteraceae families were significantly higher with 3-NOP than chloroform treatment (**Figure 3**).

**FIGURE 4:**
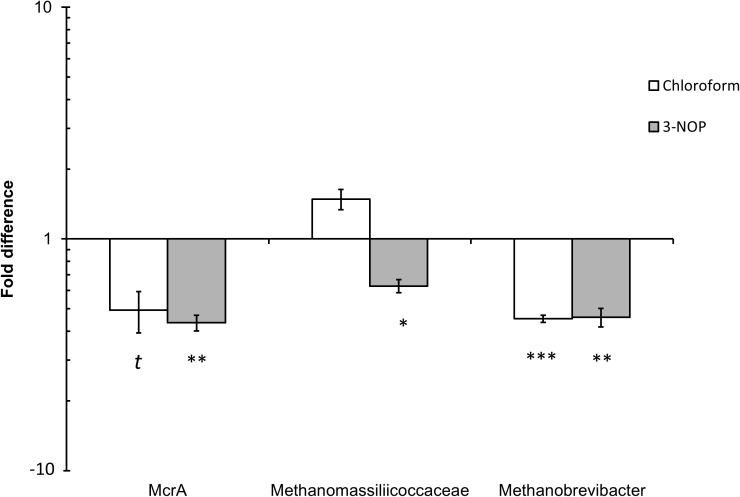
Quantitative PCR (qPCR) analysis of *mcrA* gene (methanogens), *Methanobrevibacter* spp. and Methanomassiliicoccaceae family population changes in response to chloroform or 3-NOP. Asterisks (^∗^, ^∗∗^, ^∗∗∗^) denote significant differences of treatment compared to control periods (*P* < 0.05), (*P* < 0.01) and (*P* < 0.01), respectively; the letter (t) denote a trend between control and treatment (*P* < 0.1). The y-axis denotes fold change from control period.

Quantitative PCR analysis of the effect of chloroform and 3-NOP on the abundance of methanogens, *Methanobrevibacter* spp. and Methanomassiliicoccaceae family are shown in **Figure 4**. The methanogen abundance decreased (4.9–5.6-fold) with chloroform (*P* ≤ 0.10) and 3-NOP (*P* ≤ 0.05) compared with the control period. The Methanomassiliicoccaceae family was only decreased (four-fold) with 3-NOP (*P* ≤ 0.05), while *Methanobrevibacter* was decreased (*P* ≤ 0.05) by both compounds to a similar extent (5.5-fold) compared with the control period.

## Discussion

As observed in the current study with a forage based diet, similar CH_4_ decreases of 30–37% were reported in sheep, cattle and dairy cows treated with 3-NOP and fed high quality concentrate diets (Martinez-Fernandez et al., 2014; Romero-Perez et al., 2014; Hristov et al., 2015; Haisan et al., 2016; Vyas et al., 2016a,b, 2018). Methane conversion rates, which calculate the loss of GEI to CH_4_, showed cattle on a tropical forage diet to be losing approximately 7.3% of their gross energy intake. This is a higher estimate than the energy loss as CH_4_ (6.3%) proposed by Charmley et al. (2016) for cattle fed a similar diet. This discrepancy might be due to several factors such as age, body weight, diet quality or methodology used to generate the estimate. However, our Ym values for control animals were consistent with IPCC recommendations for crop residues and by-products (5.5–7.5%) (IPCC, 2006) and Brahman cattle fed tropical grasses (5.0–7.2%) (Kennedy and Charmley, 2012). The supplementation of chloroform and 3-NOP reduced energy loss to CH_4_ by 30–40%, respectively, leading to an increase in 2.2–2.8% of energy available for capture from the feed.

Interestingly, an increase in DWG was observed for the 3-NOP treated animals, this is in agreement with published studies in dairy cows (Haisan et al., 2014; Hristov et al., 2015) which observed a body weight gain during mid-lactation. However, a recent meta-analysis (Jayanegara et al., 2017) reported that 3-NOP had a limited influence on performance of beef cattle. Despite this, a most recent feedlot study (Vyas et al., 2018) reported an improvement in feed conversion efficiency when cattle received 3-NOP mixed with a high forage or high-grain feedlot diet. Hristov et al. (2015) suggested an increase in feed digestibility might explain an increase in DWG with 3-NOP treated animals. However, in the current study rumen DM degradability (*in sacco*) decreased slightly in 3-NOP treated animals. Although the observed weight change is a promising result, it should be treated with caution due to the short trial length and the small number of animals used. Therefore, further studies evaluating the 3-NOP effects on rumen nutrient digestibility and metabolism in roughage fed ruminants would strengthen the observations from this initial investigation.

Previous studies using 3-NOP and chloroform have shown similar changes in rumen metabolism in response to CH_4_ inhibition, including a shift toward more propionic acid production and a decrease in acetate, along with an increase in branched fatty acids (Haisan et al., 2014; Romero-Perez et al., 2014; Haisan et al., 2016; Lopes et al., 2016; Martinez-Fernandez et al., 2016). In addition, CH_4_ inhibited animals usually expel more gaseous H_2_ and animals fed hay:concentrate diets were observed to expel greater amounts of H_2_ compared to those on hay only diets (Hristov et al., 2015; Martinez-Fernandez et al., 2016, 2017; Vyas et al., 2016b). The rate of H_2_ generated from slowly fermented forage diets compared with the highly fermentable forage:concentrate diets may influence the redirection of [H] thus contributing to the difference in H_2_ loss. However, surprisingly, a 30% decrease in CH_4_ by either 3-NOP or chloroform did not increase the amount of H_2_ expelled compared with the controls. This could indicate that significant amounts of [H] were redirected into reduced end-products other than CH_4_ and H_2_, such as microbial cell mass (Ungerfeld, 2015). Increases in ammonia and branched chain fatty acids may indicate changes to microbial cell mass, similar to previous observations in a study which suggested these increases were related with enhanced proteolysis and microbial protein synthesis in the rumen (Martinez-Fernandez et al., 2016). The ratio of acetate to propionate only decreased before feeding (24 h after the previous feeding) and no differences were observed at 3 h post feeding with the treatments. This discrepancy with previous studies (which mainly used concentrate supplemented diets, TMR and silages) might relate to rate of production of [H] and its use by different microbial populations when the diet is slowly fermented. Also a decrease in passage rate and other factors such as animal age and weight, quality of the hay and the low number of experimental units used might explain the lack of effect on propionate concentration at 3 h post feeding. On the other hand, an increase in concentration of acetate in both treatments raises the issue of whether reductive acetogenic bacteria may have also contributed to the redirection of [H] with the low-quality roughage diet.

Rumen methanogens can use H_2_, formate, acetate and methyl-compounds as substrates to generate CH_4_ (Seedorf et al., 2014). Until recently, CH_4_ formation by rumen methanogens was mainly attributed to the reduction of CO_2_ using H_2_ as substrate. However, a better understanding of the rumen microbiota using metagenomics and improved sequencing techniques combined with traditional culturing techniques has demonstrated that methylotrophic methanogens are present in high numbers and contribute more to rumen CH_4_ formation than initially thought (Janssen and Kirs, 2008; Poulsen et al., 2013; Seedorf et al., 2014; Martinez-Fernandez et al., 2017). Recently, Henderson et al. (2015) reported in a global rumen study that around 77% of rumen methanogens are hydrogenotrophic (mainly Methanobacteriales) and 20% methylotrophic (mainly Methanoplasmatales and *Methanosphaera* spp.). In the current study, the abundance of the total methanogen community decreased by 4–5-fold when CH_4_ was decreased by 30–37% by chloroform and 3-NOP, respectively. Regarding the hydrogenotrophic methanogens, the total abundance of *Methanobrevibacter* spp. and OTUs classified within this genus were reduced to a greater extent by the inhibitors in agreement with published *in vitro* and *in vivo* studies (Duin et al., 2016; Martinez-Fernandez et al., 2016). Although the amount of H_2_ expelled did not increase when methanogenesis was inhibited, other ruminal metabolites which are used as substrates by some methanogens, such as methylamines and alcohols, increased with both inhibitors. This might suggest that even with roughage diets methyl compounds could contribute significantly more to the CH_4_ formation than previously expected. An increase in trimethylamine was observed with both 3-NOP and chloroform, although it was greater in the 3-NOP treated-animals (three-fold increase). The decrease in abundance of the Methanomassiliicoccaceae family by 3-NOP might be related to accumulation of methylamines in the rumen fluid. However, no changes in the abundance of these methanogens were observed in the chloroform-animals although an accumulation of trimethylamine was also detected. Thus, to confirm whether this group of methanogens is more actively involved in rumen methanogenesis, gene expression should be assessed in future experiments.

Methanol is another methyl compound that can be used as a substrate by some methanogens to produce CH_4_ in the rumen. *Methanosphaera* spp. are primarily methanol users (Liu and Whitman, 2008), although a recent study identified a *Methanosphaera* spp. in the intestinal tract of macropods that can also use ethanol (Hoedt et al., 2016). Interestingly, the chloroform treatment showed a remarkable decrease in OTUs assigned to *Methanosphaera* spp., alongside a significant increase in methanol (three-fold increase) in the rumen. Another metabolite that increased in the treated-animals was the organosulfur compound DMSO_2_, which was significantly greater in the 3-NOP treated animals. DMSO_2_ is a microbial-mammalian cometabolite originating from microbial metabolism of methionine to dimethyl sulfide which is oxidized by the host tissues (He and Slupsky, 2014). Although DMSO_2_ has not been previously reported as a substrate for rumen methanogens, its precursors (such as dimethyl sulfide) are used as substrates by methanogens in anaerobic environments (Lomans et al., 1999; Scholten et al., 2003) and might contribute significantly to CH_4_ formation in the rumen. It has been demonstrated by Salsbury and Merricks (1975), that rumen bacteria are able to produce methyl sulfide from sulfur amino acids. However, the formation of DMSO_2_ in the rumen needs to be clarified.

The rumen bacterial community analysis revealed a similar profile for both treatments in line with the rumen metabolome. The decrease in Synergistetes by both inhibitors has been previously reported in ruminants treated with halogenated compounds (Denman et al., 2015; Martinez-Fernandez et al., 2016, 2017) and in low CH_4_ emitters animals (Wallace et al., 2015). It has been demonstrated previously that the growth of some Synergistetes species is optimal at a low partial pressure of H_2_, when methanogenesis is not inhibited and “interspecies hydrogen transfer” is readily occurring (Leong et al., 2016). Unlike in the previous trials, where expelled H_2_ was observed to increase and was speculated to be negatively impacting the Synergistetes, results here may point to more of a preferred direct relationship with the methanogenic archaea for inter species hydrogen transfer. The fibrolytic *Ruminococcus* species are also known to be sensitive to increased H_2_ levels (Mitsumori et al., 2012). In a dairy cow study where animals were fed a concentrate diet and supplemented with 3-NOP, expelled H_2_ was observed to increase with a decrease in *Ruminococcus* species (Lopes et al., 2016). Here using a poor quality forage diet but with redirection of [H] and low expelled H_2_ the species were not affected. In line with our observation, no apparent detrimental effects on DMIs, ruminal fermentation and rumen dry matter degradability (apart for a slight decrease with 3-NOP) were observed. Duin et al. (2016) reported that 3-NOP did not inhibit the growth of key rumen bacteria using pure culture incubations, such as *Ruminococcus albus*, *Ruminococcus flavefaciens*, *Fibrobacter succinogenes*, *Prevotella bryantii* or *Prevotella ruminicola*. Interestingly, an increase in the relative abundance of OTUs classified within Campylobacteraceae family with both compounds observed in the current study has not been reported previously and their role in the rumen needs to be clarified.

An increase of methylamines and other methylated compounds were the main alterations in the rumen metabolome but these compounds have not received much attention previously in rumen metabolism. Some studies have linked methylamines with adverse effects on health of animals fed high-grain diets (Ametaj et al., 2010; Saleem et al., 2012; Zhao et al., 2014). Formation of methyl amines from decarboxylation of some amino acids at low rumen pH and increased intestinal permeability to these compounds, may generate toxic metabolites in the liver and other organs (Saleem et al., 2012; Russell et al., 2013). However, contrary to any adverse effect of 3-NOP, some studies have reported an increase in LW or productivity in dairy cows and beef fed this compound (Haisan et al., 2014; Hristov et al., 2015; Vyas et al., 2018). In our study the accumulation of methylated compounds did not originate from a dietary excess, but, rather, arose from an inhibition of methanogenesis using a low-quality diet at a physiological rumen pH. Thus, the effect of methylamines in ruminant health warrants further investigation as we speculate that the accumulation of methyl compounds in the rumen of animals fed with low-quality diets might have a positive effect on the health of the host by acting as methyl donor compounds to reduce oxidative stress (Alirezaei et al., 2014).

## Conclusion

Despite the different mode of action of 3-NOP on methanogens, inhibition of methanogenesis by 3-NOP and chloroform resulted in similar responses in metabolism and microbial community structure in the rumen. It also shows that the use of 3-NOP on a forage diet produces similar levels of CH_4_ inhibition compared with previous studies based on concentrate supplemented diets. We hypothesize that the rumen response to direct inhibition of methanogenesis is reflected in a redirection of [H] away from CH_4_ formation to other reduced end-products that may benefit the host animal. Therefore results from previous publications using chloroform as an inhibitor of methanogenesis may be useful in predicting responses to 3-NOP with regard to the rumen microbiota and fermentation. However, despite the similarities observed with both compounds, chloroform cannot be used under farming conditions due to its toxicity and environmental impacts. Finally, the promising increase in body weight observed with 3-NOP treatment should be confirmed with further research using greater numbers of animals and focussed on animal productivity and metabolism.

## Author Contributions

CM, SED, SD, MK, and GM-F conceived and designed the experiments and analytical approaches. GM-F performed the animal trial. GM-F and HS analyzed the biological samples. SED, HS, and GM-F analyzed the data. CM, SED, and GM-F wrote the manuscript. All authors agreed to be accountable for all aspects of the work.

## Conflict of Interest Statement

SD and MK declared that were employed by DSM Nutritional Products, which is the owner and patented 3-NOP. The remaining authors declared that the research was conducted in the absence of any commercial or financial relationships that could be construed as a potential conflict of interest.
