# Supplementary material for: 3-NOP vs. Halogenated Compound: Methane Production, Ruminal Fermentation and Microbial Community Response in Forage Fed Cattle
Source: Front Microbiol. 2018 Aug 7;9:1582. doi: 10.3389/fmicb.2018.01582 (PMC6090035; doi:10.3389/fmicb.2018.01582)
Supplement: Supplementary file 1 [file Data_Sheet_1.docx]

***Supplementary Material***

**3-NOP vs Halogenated compound: Methane production, ruminal fermentation and microbial community response in forage fed cattle**

**Gonzalo Martinez-Fernandez^1^*, Stephane Duval^2^, Maik Kindermann^3^, Horst Joachim Schirra^4^, Stuart E. Denman^1^ and Christopher S. McSweeney^1^**

^1^ CSIRO, Agriculture and Food, Queensland Bioscience Precinct, St Lucia, QLD, Australia

^2^ Research Centre for Animal Nutrition and Health, DSM Nutritional Products, Saint-Louis Cedex, France

^3^ Animal Nutrition and Health, DSM Nutritional Products, Basel, Switzerland.

^4^ The University of Queensland, Centre for Advanced Imaging, Brisbane, QLD, Australia

*** Correspondence:** Gonzalo Martinez-Fernandez Email:gonzalo.martinezfernandez@csiro.au

1. **Supplementary Tables and Figures**
   1. **Supplementary Tables**

**Supplementary Table 1.** Resonance assignments for key metabolites.

| Metabolites^a^ | Moieties: δ ^1^H (ppm)/ δ ^13^C (ppm) |
| --- | --- |
| 2-aminobutyrate | γCH_3_: 0.954(t)^b^ |
| 3-phenylpropionate | δCH: 7.30(m)/131.1, εCH: 7.36(m)/131.2, ζCH: 7.26(m)/128.9, βCH_2_: 2.885(dd)/34.6, αCH_2_: 2.48(dd)/41.8 |
| 5-aminopentanoate | αCH_2_: 2.224(t)/39.5, βCH_2_: 1.62(m)/25.2, γCH_2_: 1.65(m)/29.4, δCH_2_: 3.006(dd)/42.2 |
| acetate | αCH_3_: 1.91(s)/26.0 |
| acetone | CH_3_: 2.22(s)^b^ |
| alanine | βCH_3_: 1.47(d)/19.0, αCH: 3.78(q)/53.4 |
| aspartate | αCH: 3.89(dd)/55.1, βCH_2_ (x): 2.80(dd)/39.4, βCH_2_ (y): 2.674(dd)/39.4 |
| benzoate | δCH: 7.865(m)^b^, εCH: 7.477(m)^b^, ζCH: 7.55(m)^b^ |
| betaine | N-CH_3_: 3.255(s)/56.1, αCH_2_: 3.892(s)/68.9 |
| butyrate | γCH_3_: 0.885(t)/15.9, βCH_2_: 1.55(m)/22.1, αCH_2_: 2.148(t)/42.3 |
| dimethylamine | N(CH_3_)_2_: 2.708(s)/37.4 |
| dimethylsulfone | CH_3_: 3.144(s)/44.3 |
| ethanol | CH_3_: 1.17(t)^b^, CH_2_: 3.65(q)^b^ |
| formate | CH: 8.45(s)^b^ |
| α-glucose | 1β-CH: 4.641(d)^b^, 1α-CH: 5.23(d)^b^ |
| glutamate | γCH_2_: 2.34(m)/36.1, αCH: 3.75(dd)/57.5 |
| glycine | αCH_2_: 3.55(s)/44.4 |
| isobutyrate | βCH_3_: 1.05(d)/22.1, αCH: 2.38(m)/39.6 |
| isovalerate | γCH_3_: 0.90(d)/24.6, βCH: 1.94(m)/28.9, αCH_2_: 2.05(t)/49.9 |
| lactate | βCH_3_: 1.32(d)^b^, αCH: 4.11(q)^b^ |
| maltose | 1.1α-CH: 5.40(dd)^b^, 2.1α-CH: *5.23*(d)^b^, 2.1β-CH: *4.642*(d)^b^ |
| medium-chain FAs^c^ | CH_3_: 0.85(t)/14.4, CH_2_: 1.27(m)31.17 |
| methanol | CH_3_: 3.35(s)/51.7 |
| methylamine | N-CH_3_: 2.594(s)/27.6 |
| N,N-dimethylglycine | N-CH_3_: 2.914(s)/46.3, αCH_2_: 3.714(s)/62.7 |
| phenylacetate | ζCH: 7.30(m)/129.2, δCH: 7.30(m)/131.9, εCH: 7.38(m)/131.2, αCH_2_: 3.53(s)/47.1 |
| propionate | βCH_3_: 1.046(t)/12.9, αCH_2_: 2.171(q)/33.4 |
| trimethylamine | N(CH_3_)_3_: 2.866(s)/47.4 |
| uracil | 6-CH: 7.532(d)^b^, 5-CH: 5.794(d)^b^ |
| valerate | δCH_3_: 0.88(t)/15.9, γCH_2_: 1.29(m)/24.7, βCH_2_: 1.516(m)/30.8, αCH_2_: 2.17(t)/40.2 |
| valine | γ^y^CH_3_: 0.98(d)^b^, αCH: 3.60(d)^b^ |

The resonance assignment of metabolites was based on Chenomx NMR Suite 8.2 as well as 1D and 2D NMR experiments. ^1^H and ^13^C chemical shifts of metabolites obtained from ^1^H-^13^C heteronuclear experiments are presented. Key: s, singlet; d, doublet; dd, doublet of doublet; t, triplet; q, quartet; m, multiplet; b, broad.

^a^ The assignments of most metabolites are equivalent to Metabolomics Standard Initiative level 2 (putatively annotated compounds, *i.e.* assignments based upon physicochemical properties and/or spectral similarity with public/commercial spectral libraries but no internal standard used).

^b^ Corresponding ^13^C chemical shifts were not detected by ^1^H-^13^C heteronuclear experiments.

^c^ analysed as caproate during quantification.

**Supplementary Table 2.** Figures of merit of the principal components analysis (PCA) models of ruminal fluid metabolites.

| Experiment | Groups | Method | Scaling | Normalisation | *A*^a^ | *N*^b^ | *k^c^* | *R^2^X*^d^ | *Q^2^* |
| --- | --- | --- | --- | --- | --- | --- | --- | --- | --- |
| Untargeted analysis | All | PCA | Pareto | Total intensity | 2 | 16 | 9300 | 0.47 | 0.123 |
|  | CHCl_3_ & CHCl_3_ controls | PCA | Pareto | Total intensity | 2 | 8 | 9300 | 0.629 | -0.00926 |
|  | NOP & NOP controls | PCA | Pareto | Total intensity | 2 | 8 | 9300 | 0.603 | -0.0241 |
|  | CHCl_3_ & NOP | PCA | Pareto | Total intensity | 2 | 8 | 9300 | 0.617 | 0.0857 |
| Targeted analysis | All | PCA | Pareto | None^e^ | 2 | 16 | 29 | 0.887 | 0.574 |
|  | CHCl_3_ & CHCl_3_ controls | PCA | Pareto | None^e^ | 2 | 8 | 29 | 0.903 | 0.47 |
|  | NOP & NOP controls | PCA | Pareto | None^e^ | 3 | 8 | 29 | 0.962 | 0.663 |
|  | CHCl_3_ & NOP | PCA | Pareto | None^e^ | 3 | 8 | 29 | 0.966 | 0.671 |

^a^ Number of principal components

^b^ Number of samples

^c^ Number of *X* variables = number of spectral integral regions (“buckets”) in the untargeted analysis, and number of quantified metabolites in the targeted analysis.

^d^ *R^2^X* is the fraction of the sum of squares for the selected component representing the variance of *X* variables, and *Q^2^* is the predictive ability parameter of the model, which is estimated by cross-validation. The two negative *Q^2^* values in the table (shaded grey) denote weak PCA models.

^e^ Absolute molar concentrations were used in all targeted models.

**Supplementary Table 3.** 3-NOP effects on ruminal fermentation parameters in steers prior-feeding (24 h after offering hay).

|  | Control | 3-NOP | SEM^a^ | *P-value* |
| --- | --- | --- | --- | --- |
| NH_3_-N (mg/100 mL) | 14.7 | 16.8 | 1.70 | 0.508 |
| Total SCFA, (mM) | 92.4 | 99.4 | 4.93 | 0.018 |
| Individual SCFA (mol/100 mol) | 14.7 | 16.8 |  |  |
| Acetate | 74.4 | 71.0 | 0.43 | 0.009 |
| Propionate | 15.8 | 17.3 | 0.29 | 0.146 |
| i-Butyrate | 0.88 | 1.20 | 0.05 | 0.102 |
| Butyrate | 6.92 | 7.49 | 0.18 | 0.073 |
| i-Valerate | 1.06 | 1.87 | 0.07 | 0.001 |
| Valerate | 0.85 | 0.98 | 0.01 | 0.059 |
| Caproate | 0.14 | 0.22 | 0.01 | 0.023 |
| A:P | 4.71 | 4.14 | 0.09 | 0.071 |

^a^ SEM, standard error of the mean

**Supplementary Table 4.** Chloroform effects on ruminal fermentation parameters in steers prior-feeding (24 h after offering hay).

|  | Control | Chloroform | SEM^a^ | *P-value* |
| --- | --- | --- | --- | --- |
| NH_3_-N (mg/100 mL) | 8.81 | 20.9 | 1.17 | 0.031 |
| Total SCFA, (mM) | 99.3 | 108.1 | 4.56 | 0.128 |
| Individual SCFA (mol/100 mol) |  |  |  |  |
| Acetate | 71.9 | 70.6 | 0.39 | 0.261 |
| Propionate | 16.0 | 17.6 | 0.40 | 0.031 |
| i-Butyrate | 0.79 | 1.10 | 0.10 | 0.196 |
| Butyrate | 8.85 | 7.34 | 0.28 | 0.106 |
| i-Valerate | 1.24 | 1.80 | 0.04 | 0.061 |
| Valerate | 0.87 | 1.17 | 0.06 | 0.009 |
| Caproate | 0.32 | 0.36 | 0.06 | 0.360 |
| A:P | 4.52 | 4.02 | 0.12 | 0.045 |

^a^ SEM, standard error of the mean

- 1. **Supplementary Figures**

**
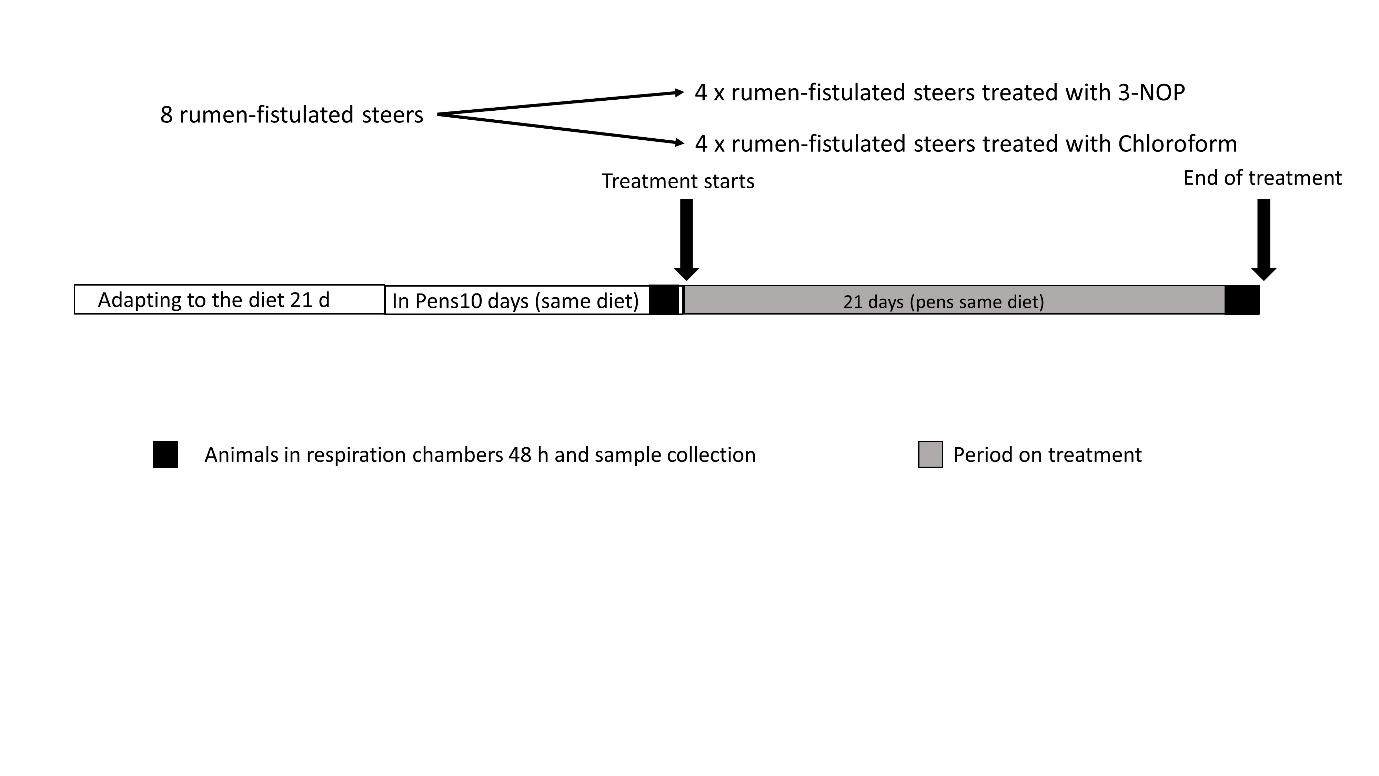
**

**Supplementary Figure 1.** Experimental timeline.


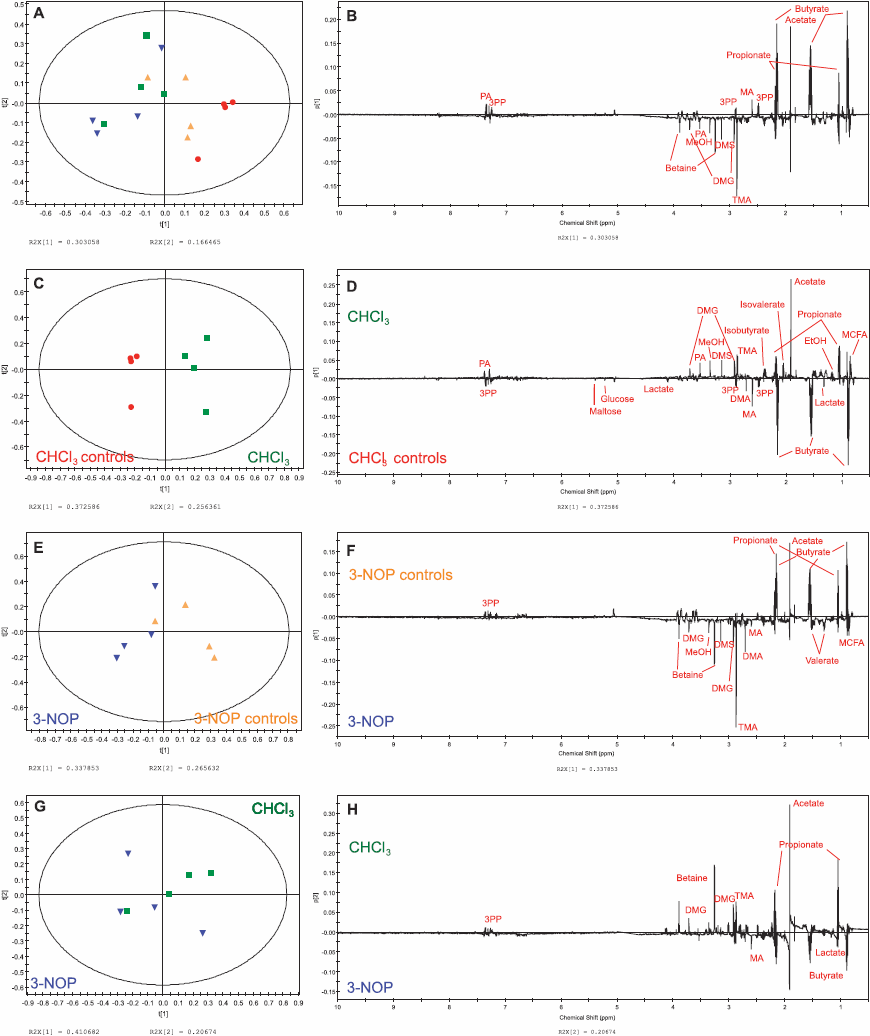


**Supplementary Figure 2.** Initial untargeted principal components analysis (PCA) of ruminal fluid samples. 1D NOESY NMR spectra were used. t[1] and t[2] are the first and second PCA components, respectively. The percentage of variation explained by each component is shown in brackets. (A) Scores plot of the PCA containing all samples. Red circles: chloroform (CHCl_3_) controls, green squares: chloroform (CHCl_3_)-treated samples, orange triangles: 3-NOP controls, blue inverted triangles: 3-NOP-treated samples. (B) 1D loadings plot of the PCA depicted in (A). The loadings coefficients are plotted against the chemical shift of the variable in the NMR spectrum, thus appearing like a 1D NMR spectrum. Positive loadings indicate metabolites correlated with controls and negative loadings indicate metabolites correlated with treated samples. (C, D): Scores (C) and loadings plot (D) of a PCA containing only samples from the CHCl_3_ trial. Positive and negative loadings indicate metabolites correlated with the annotated sample group, respectively. (E, F): Scores (E) and loadings plot (F) of a PCA containing only samples from the 3-NOP trial. Positive and negative loadings indicate metabolites correlated with the annotated sample group, respectively. (G, H): Scores (G) and loadings plot (H) of a PCA comparing CHCl_3_- and 3-NOP-treated samples. Positive and negative loadings indicate metabolites correlated with the annotated sample group, respectively. The figures of merit of these models are listed in S Table 2. 3PP: 3-phenylpropionate, DMA: dimethylamine, DMG: N,N-dimethylglycine, DMS: dimethylsulfone, EtOH: ethanol, MA: methylamine, MCFA: medium-chain fatty acids, MeOH: methanol, PA: phenylacetate, TMA: trimethylamine.


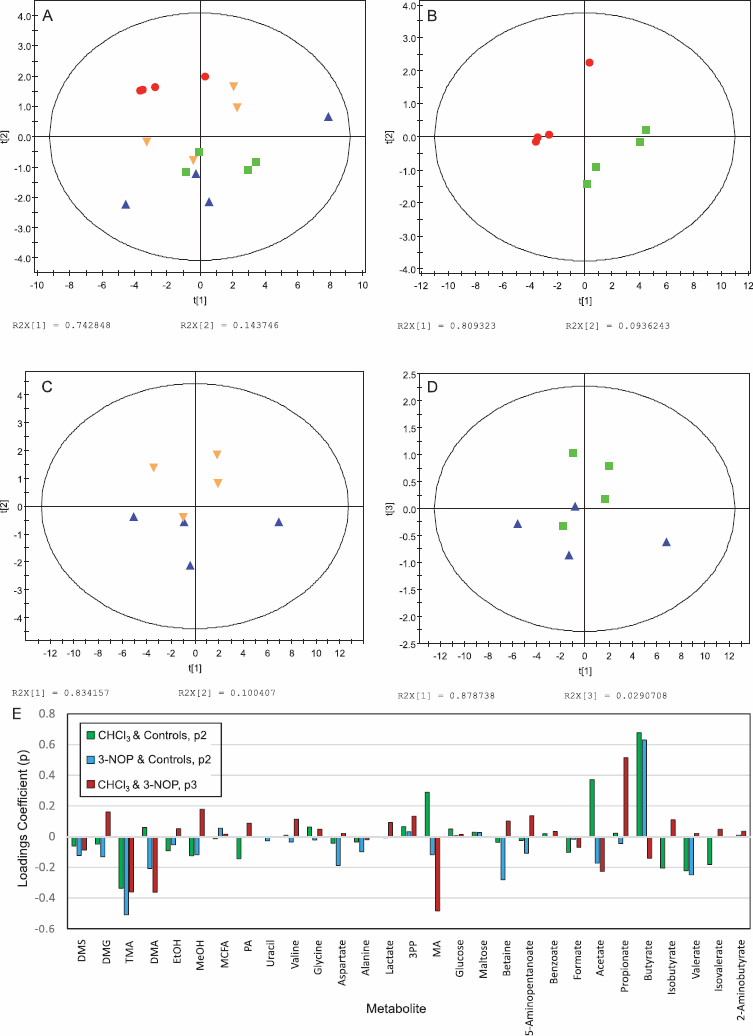


**Supplementary Figure 3.** Targeted PCA of ruminal fluid samples. The models are based on the absolute molar concentrations of selected metabolites identified in Supplementary Figure 1 and quantified in the 1D NOESY NMR spectra. t[1] and t[2] are the first and second PCA components, respectively. The percentage of variation explained by each component is shown in brackets. (A) Scores plot of the PCA containing all samples. Red circles: chloroform (CHCl_3_) controls, green squares: chloroform (CHCl_3_)-treated samples, orange triangles: 3-NOP controls, blue inverted triangles: 3-NOP-treated samples. (B): Scores of a PCA containing only samples from the CHCl_3_ trial. (C): Scores plot of a PCA containing only samples from the 3-NOP trial. (D): Scores plot of a PCA comparing CHCl3- and 3-NOP-treated samples. (E) Loadings coefficients for the three PCA models in (B-D). Green: CHCl_3_ model, blue: 3-NOP model, brown: comparison of CHCl_3_- and 3-NOP-treated samples. The figures of merit of these models are listed in Supplementary Table 2. Abbreviations are as in Supplementary Figure 2.

**
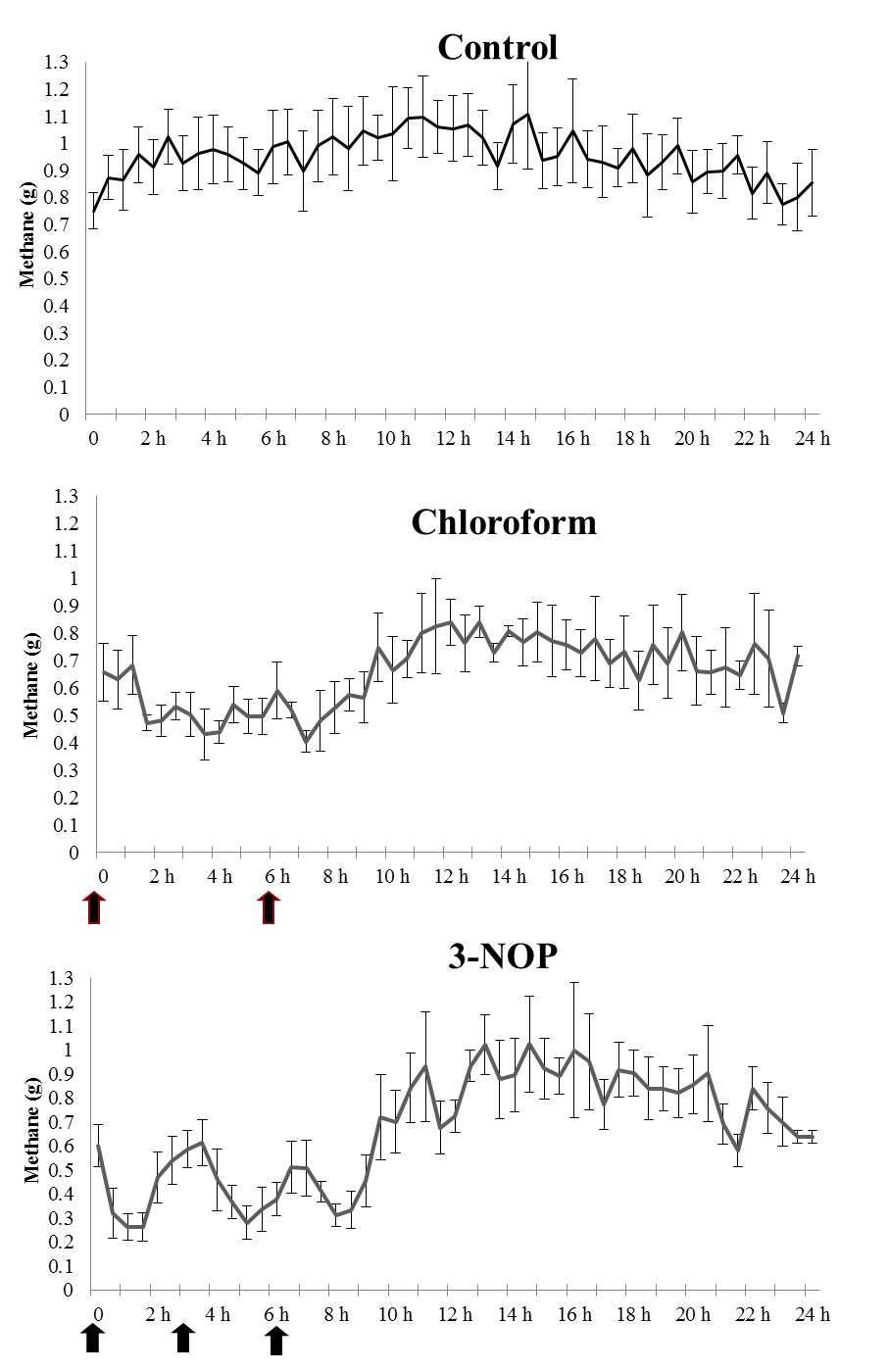
**

**Supplementary Figure 4.** Methane emissions pattern over 24 h of steers during control period (no treatment), chloroform and 3-NOP treatments. Treatment provided.


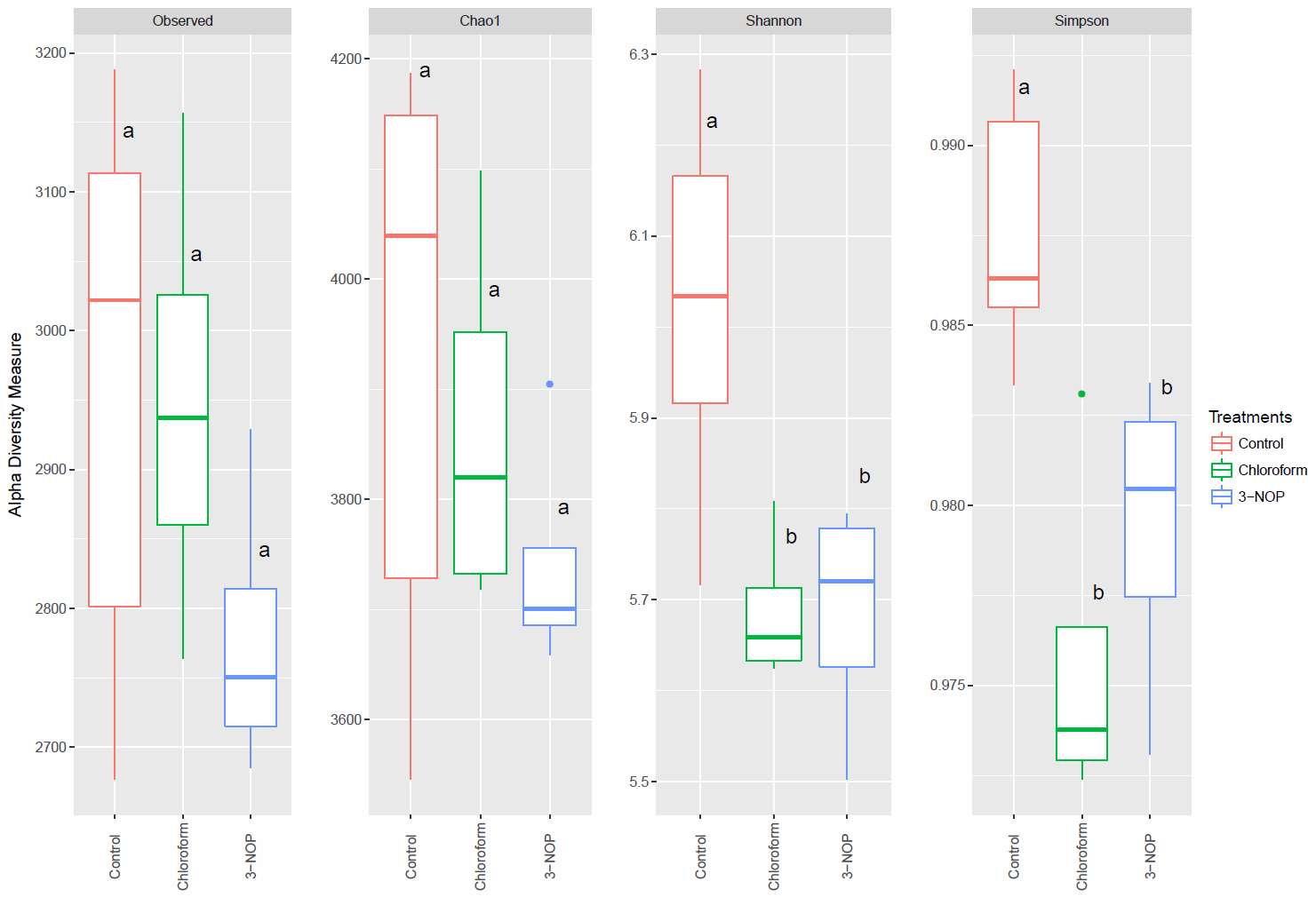


**Supplementary Figure 5.** Alpha diversity measures for rumen microbiomes at control, chloroform and 3-NOP illustrating the total observed taxonomic units (Observed) and the Shannon diversity index (Shannon). Boxplots indicate variance within the sampled animals with the box boundaries showing the first and third quartiles, the median value indicated as a horizontal line and the whiskers extend to 1.5 times the interquartile range. ^a-b^Letters denote significant differences between treatments for each Alpha diversity measure, boxplots that do not share the same letter are significantly different from each other (*P* < 0.05).


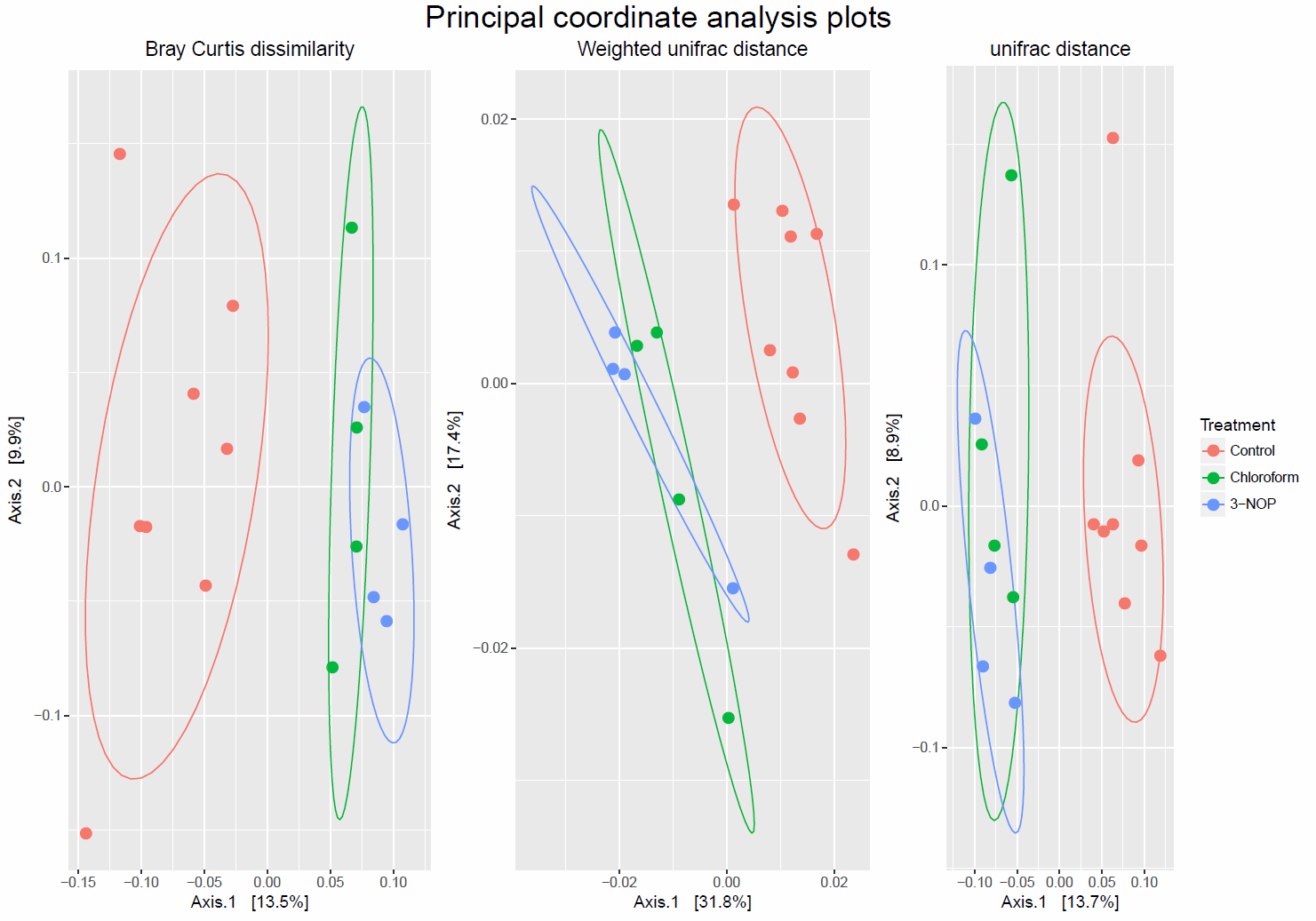


**Supplementary Figure 6.** Principle Coordinate Analysis comparing changes in microbial OTU classification based on Bray Curtis dissimilarity, weighted and unweighted Unifrac calculations for control (red), chloroform (green) and 3-NOP (blue).


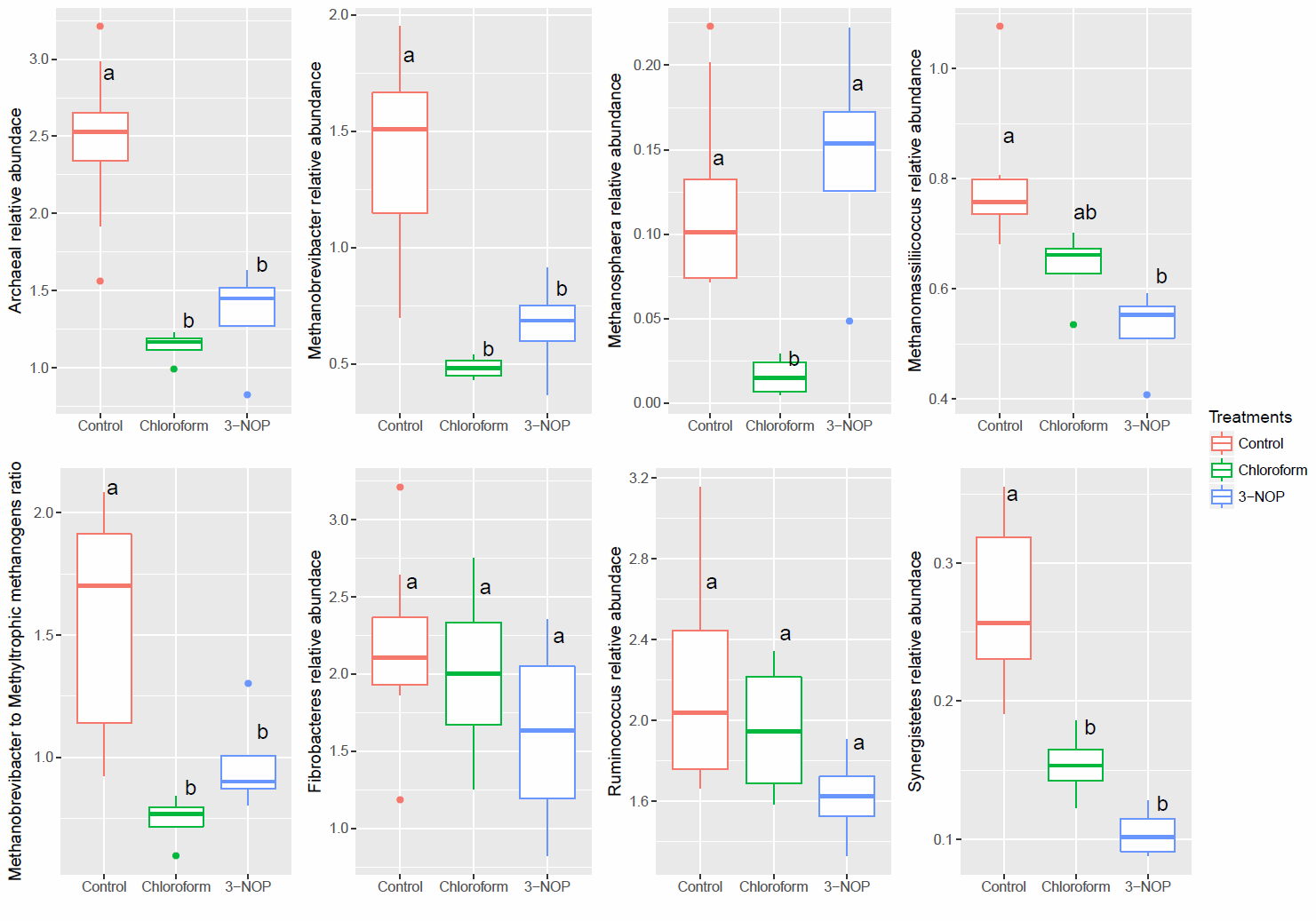


**Supplementary Figure 7.** Microbial ratios (Archaea:Bacteria, *Methanobrevibacter*:Bacteria, *Methanosphaera:*Bacteria, *Methathanomassiliicoccus:*Bacteria, *Methanobrevibacter*:Methylotrophic methanogens, *Fibrobacteres*:Bacteria, *Ruminococcus*: Bacteria, and Synergistetes:Bacteria,) for controls, chloroform and 3-NOP in steers fed a hay diet. ^a-b^Letters denote significant differences between treatments for each microbial ratio, boxplots that do not share the same letter are significantly different from each other (*P* < 0.05).
